# Development and evaluation of a standardized technique to assess teat skin temperature of dairy cows using infrared thermography

**DOI:** 10.3168/jdsc.2021-0181

**Published:** 2022-02-10

**Authors:** C. DiLeo, P.S. Basran, I.R. Porter, M. Wieland

**Affiliations:** 1 College of Veterinary Medicine, Cornell University, Ithaca, NY 14853; 2 Department of Clinical Science, College of Veterinary Medicine, Cornell University, Ithaca, NY 14853; 3 Department of Population Medicine and Diagnostic Sciences, College of Veterinary Medicine, Cornell University, Ithaca, NY 14853

## Abstract

Much research has been done to develop methods to assess dimensional teat traits in dairy cows. In contrast, diagnostic techniques to reliably assess the circulatory system of teats are limited. Infrared thermography facilitates measurements of skin temperature and could be used to detect physiological and pathological changes to the teat tissue associated with machine milking, as temperature reflects the underlying blood circulation and tissue metabolism. Our objective was to develop and evaluate a scanning technique to quantify teat skin temperature in dairy cows using infrared thermography. Using a portable thermography camera, 2 operators obtained duplicate scans of both hind teats from 20 Holstein cows, resulting in 80 thermographic images (20 cows × 2 operators × 2 images). Average teat skin temperatures at the proximal, middle, and distal teat aspects were determined. We used Pearson correlation coefficients (r), intraclass correlation coefficients (ICC), and concordance correlation coefficients (CCC) to assess interoperator reproducibility (i.e., agreement between measurements performed by different operators) and intraoperator repeatability (i.e., agreement between measurements performed by the same operator). Pearson correlation coefficients revealed a very strong correlation for measurements at the proximal, middle, and distal aspects of the teat, respectively, between operators (r ≥0.95) and duplicate scans (r ≥0.94) within operators. Intraclass correlation coefficients and CCC indicated excellent interoperator reproducibility (ICC ≥0.95, CCC ≥0.95) and excellent intraoperator repeatability (ICC ≥0.94, CCC ≥0.94), respectively, for measurements at all 3 aspects. Least squares means (95% confidence interval) for average teat skin temperatures at the proximal, middle, and distal teat aspects, respectively, were 33.2 (32.6–33.8), 32.4 (31.5–33.2), and 30.9 (29.8–32.0) °C for operator 1, and 33.2 (32.6–33.8), 32.4 (31.6–33.3), and 31.0 (29.9–32.0) °C for operator 2. Average temperatures between duplicate scans within operators at the proximal, middle, and distal aspects, respectively, were 33.3 (32.7–33.9), 32.5 (31.7–33.3), and 31.0 (29.9–32.1) °C for the first scan and 33.2 (32.6–33.8), 32.3 (31.5–33.1), and 30.8 (29.7–31.9) °C for the second scan. We conclude that infrared thermography facilitates precise measurements of skin temperatures of cows' hind teats.

## Highlights

• Infrared thermography (IRT) can be used to measure surface temperatures without direct contact.
• IRT may be useful to indirectly estimate teat blood circulation by assessing teat skin temperature.
• Precision was assessed by means of interoperator reproducibility and intraoperator repeatability of temperature measurements of hind teats from 20 cows.
• Agreement results suggest that IRT facilitates precise measurements of skin temperature of cows' hind teats.

## Graphical Abstract

**Figure:**
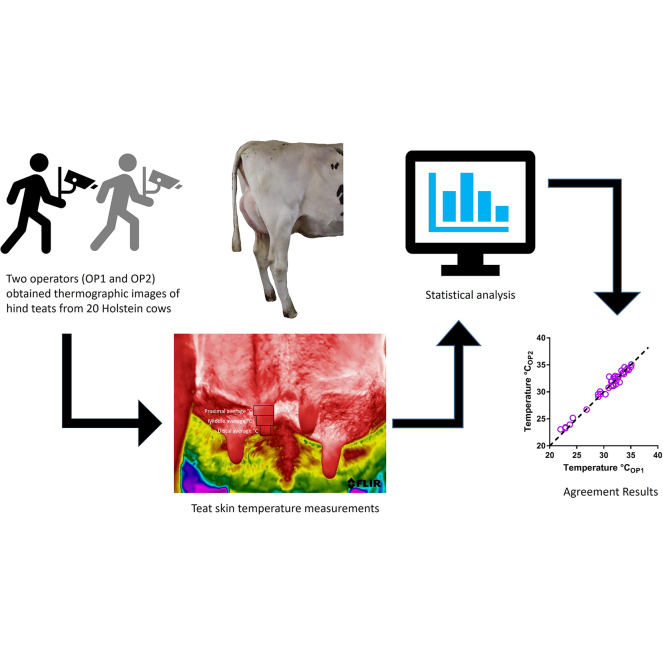
**Summary:** Infrared thermography (IRT) facilitates contactless measurements of surface temperature and could be used to assess teat blood circulation of dairy cows by assessing teat skin temperature. Here, we describe the development and evaluation of a scanning technique to quantify teat skin temperature with IRT. Two operators obtained duplicate images of hind teats with a portable thermography camera. One investigator divided the teats into proximal, middle, and distal segments and recorded average temperature for each segment. We assessed the precision of measurements by means of interoperator reproducibility and intraoperator repeatability; agreement results were excellent for both. We conclude that precise measurements of teat skin temperature can be obtained using IRT.

Mechanical forces of machine milking can change the teat morphology (Odorcic et al., 2020). During milking, differences in pressure between the liner barrel and pulsation chamber (i.e., the space between the liner and teat cup shell) exert a force on the liner, causing it to either collapse or open. A closed liner compresses the teat and closes the teat orifice, halting milk flow (i.e., rest phase), while an open liner exposes the teat end to vacuum creating milk flow (i.e., milk phase). The International Organization for Standardization standard 3918 (ISO, 2007) defines the liner positions in each cycle as opening (a-phase), open (b-phase), closing (c-phase), and closed (d-phase). During the b-phase, vacuum applied to the teat increases transmural pressure in vessels, leading to congestion and edema, thickening the teat tissue (Schalm et al., 1971). In a paired ultrasound and histologic assessment of teats directly after milking, Stauffer et al. (2021) found the origin of the increase in diameter of the thick-walled veins, with the changes in these veins being restricted to the luminal aspect of the vessels (i.e., vascular congestion) rather than accumulation of extravascular fluid (i.e., edema). When the teat is congested, the skin limits its lateral expansion and the teat canal narrows (Penry et al., 2017). Conditions that can precipitate an increase in teat thickness and narrowed canal are a shortened d-phase, overmilking, or high vacuum paired with a longer b-phase and a mouthpiece connected to vacuum (Upton et al., 2016; Penry et al., 2017; Odorcic et al., 2020). An increase in teat tissue thickness is associated with microbial colonization of the teat canal (Zecconi et al., 1992) and increased susceptibility to new IMI (Zecconi et al., 1996), making congestion an important parameter to monitor.

To better understand the effect of machine milking on the occurrence of IMI, it is necessary to use technologies that can efficiently and precisely monitor teat tissue changes. Current techniques include the use of a cutimeter (Hamann and Mein, 1988), B-mode ultrasonography (Bruckmaier and Blum, 1992), and power Doppler ultrasonography (Wieland et al., 2020). A cutimeter or B-mode ultrasonography indirectly measures congestion and edema by measuring teat tissue thickness and teat wall diameter, respectively. Power Doppler has an advantage because it directly measures blood flow patterns. However, using a cutimeter and ultrasound require direct contact with the teat—direct contact for the cutimeter and via lube or a cup filled with water for ultrasound. Direct contact with the teat stimulates teat muscle contractions, which decrease arterial blood flow in the teat and affect teat dimensions (Maltz et al., 2000). This may increase the variation in measurements, hampering the ability to make causal inferences about, for example, the effect of machine milking on tissue condition and blood circulation of the teat. Additionally, Wieland et al. (2019) found poor reproducibility between operators, demonstrating the need to refine the power Doppler technique before it can be widely used.

Another method to detect changes in teat tissue after milking is infrared thermography. The principle underlying thermography is that all bodies emit electromagnetic radiation. Based on the Stefan-Boltzmann law, the amount of energy that a body emits per unit time in a given area (or its emissive power) is a function of the body's emissivity and its temperature. The perfect emitter, a black body, absorbs and emits all radiation regardless of wavelength or direction, and its emissivity is 1 (i.e., Max Planck's law). A teat's emissivity is around 0.95 to 0.98 (Paulrud and Rasmussen, 2004). Temperature is captured by thermographic cameras that use a sensor to collect data on the radiation the teat emits. Thermography provides a synopsis of the physiological changes that occur in the teat as a result of machine milking. Thermography does not require contact with the teat, leading to a minimal effect of measurement on teat tissue dynamics and minimizing cow handling stress. Paulrud et al. (2005) used thermography to detect effects of milking liner structure and overmilking on teat tissue recovery and found that increases in teat temperature during milking were dependent on liner type. Similarly, Barkova et al. (2019) found differences in temperature patterns resulting from differences in milking machines. Tangorra et al. (2019) tested whether thermography could be used to color score teats and found low sensitivity and specificity for this application. These studies demonstrate an opportunity to apply thermography to assess teat tissue changes; however, relatively few studies have assessed the suitability of thermography to detect changes in teat tissue as a result of machine milking. Our study builds on the existing applications of thermography and addresses the paucity of research on its suitability to monitor machine milking–induced changes in teat tissue. The objective of this study was to determine the precision of thermographic measurements, represented by interoperator reproducibility and intraoperator repeatability of temperature averages in the proximal, middle, and distal regions of the teat. We hypothesized that precise measurements of teat skin temperature could be obtained using infrared thermography.

The Cornell University Institutional Animal Care and Use Committee reviewed and approved all procedures (protocol no. 2021-0009). We conducted this study in March 2021 at the Teaching Dairy Barn of the College of Veterinary Medicine, Cornell University (Ithaca, NY). The farm milks approximately 160 cows that are housed year-round in 2 freestall pens, bedded with recycled sand, and fed a TMR consistent with NRC (2001) requirements. Cows are milked 3 times a day in a double-10 parallel milking parlor. The average production level of the herd was 40.7 kg/cow per day during the study period.

Eligible cows had to be at least 50 DIM, free of clinical mastitis for the last 4 wk, and free of udder abnormalities such as nonlactating quarters or teat injuries. Because we anticipated that cows had to stand for approximately 1.5 h, eligible cows were also required to be free of lameness and must have had a record of normal handling ease. We selected a convenience sample of 20 cows that were in their first (n = 12), second (n = 5), third (n = 1), fourth (n = 1), or sixth (n = 1) lactation and between 51 and 350 DIM. Thermographic images of both hind teats were obtained using a portable thermography camera (FLIR T530, Teledyne FLIR LLC) by 2 operators (C.D. and M.W.) in the milking parlor between the morning and noon milking sessions on 2 subsequent days. Before the study, the camera was calibrated, and the imaging modes were identified and kept consistent throughout the trial. These included the autofocus function and the “laser” option, where the focus is based on a laser distance. The laser distance meter was enabled to automatically determine the object distance. The reflective temperature was kept at 20°C and the emissivity at 0.95. The atmospheric temperature and relative humidity were retrieved from the local weather station and were as follows: d 1, 12°C, 52%; d 2, 10°C, 79%.

Images were taken from the caudal aspect of the udder in a caudo-to-cranial direction from a distance of approximately 0.5 m. To facilitate assessment of intraoperator repeatability, we obtained duplicate images such that each operator captured 2 images sequentially from each cow. All scans were labeled with the cow identification number, operator initials, and sequence using the note function and subsequently stored on the integrated flash drive. The sample size of 20 cows, resulting in 40 teat observations, was based on McBride (2005), who recommended a sample of ≥25 for assessment of concordance correlation coefficient (**CCC**).

We used the adjunct software program (FLIR Tools, Teledyne FLIR LLC) to conduct measurements of teat skin surface temperatures at the (1) proximal, (2) middle, and (3) distal aspects of the left and right hind teats, respectively. The software program provides different measurement tools to calculate the temperature at a single location (i.e., spotmeter) or the average, minimum, and maximum temperatures of regions of interest outlined by different geometric structures (i.e., line, rectangle, ellipse). We determined the average temperatures of the teat skin of 3 regions of interest using the rectangle tool. Our reasoning for determining average temperatures rather than a single temperature measurement was based on the attempt to decrease measurement error. Because we aimed to segment the teat into 3 anatomical regions of interest as described previously (Paulrud et al., 2005; Barkova et al., 2019; Tangorra et al., 2019), we obtained measurements from the proximal, middle, and distal aspects of the teat (Figure 1). One trained investigator (C.D.) consistently performed the following steps according to a standard operating procedure. First, the proximal and distal boundaries of the teat were defined. For this purpose, a rectangle was drawn from the teat base to the teat end. Subsequently, 3 separate rectangles of equal height were drawn at the proximal, middle, and distal thirds of the teat. The width of the teat at each aspect determined the width of the rectangle such that the corners did not exceed the boundary of the teat demarcation. Finally, the average temperatures of each region of interest outlined with the rectangle were obtained. During measurements, the notes including information on cow identification number, operator initials, and sequence remained obscured.

**Figure 1:**
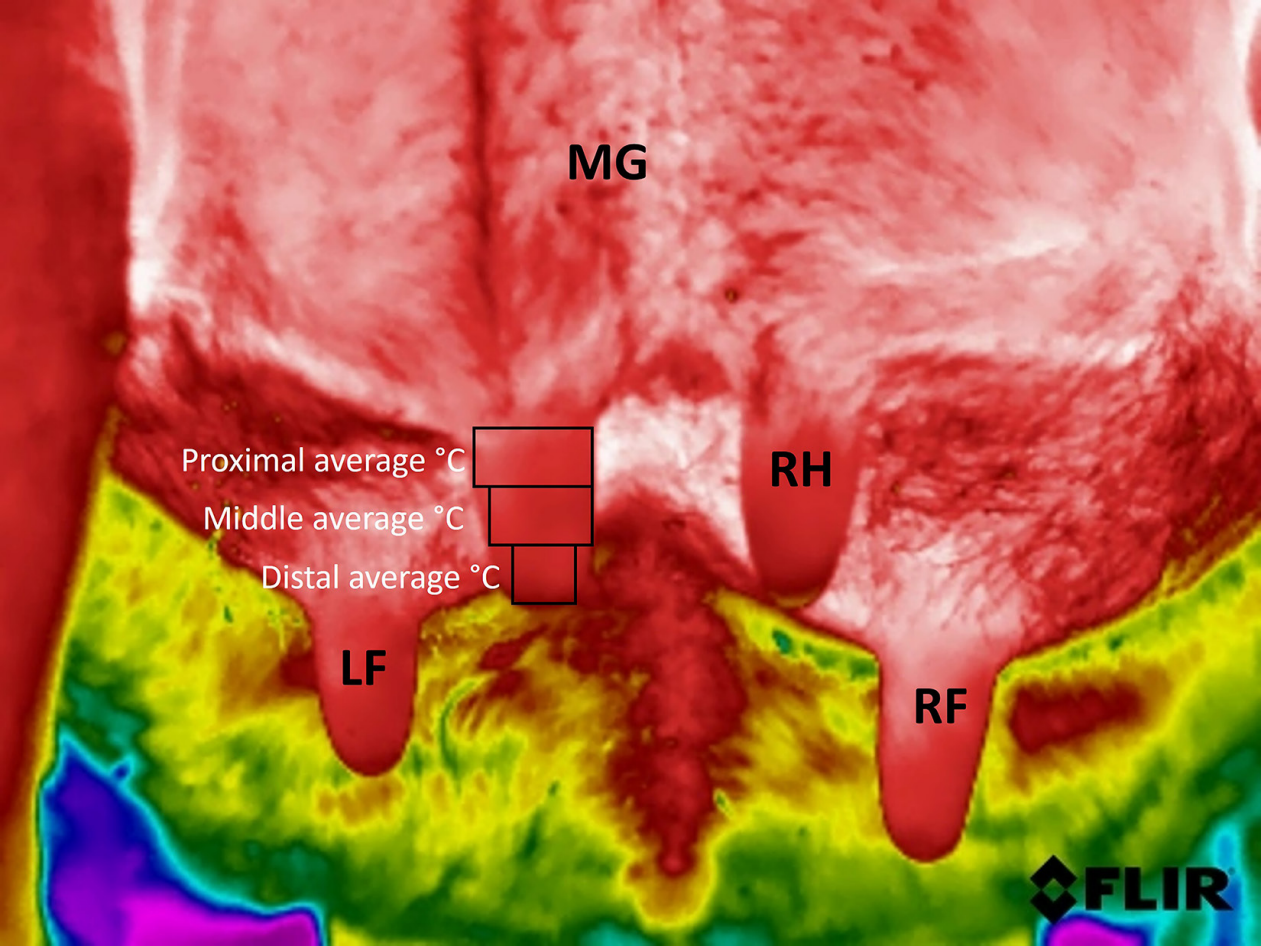
Thermographic image showing aspects of the mammary gland (MG) and the left hind, left front (LF), right front (RF), and right hind (RH) teats of a cow. The proximal, middle, and distal aspects of the teat used to calculate the average temperatures of the 3 different regions of interest are depicted by the 3 rectangles overlying the left hind teat.

We maintained data in Excel (2019, Microsoft Corp.) and performed statistical analyses using R statistical software (https://www.r-project.org/). We assessed interoperator reproducibility using only the first data set (no duplicate scans) by calculating Pearson correlation coefficients (r), intraclass correlation coefficients [**ICC** (95% CI)] according to Shrout and Fleiss (1979) using the “psych” package (Revelle, 2020), and CCC (95% CI) according to Lin (1989) for nonrepeated measurements as described by Carrasco et al. (2013) using the “cccrm” package (Carrasco and Martinez, 2021). We evaluated intraoperator repeatability of duplicate scans by Pearson correlation coefficients, ICC (95% CI) with the “psych” package (Revelle, 2020), and CCC (95% CI) for nonlongitudinal repeated measurements as described by Carrasco et al. (2013) with the “cccrm” package (Carrasco and Martinez, 2021). Pearson correlation coefficients were classified according to Schober et al. (2018) as negligible (0.00 to 0.09), weak (0.10 to 0.39), moderate (0.40 to 0.69), strong (0.70 to 0.89), and very strong (0.90 to 1.00). Results of ICC and CCC were classified as poor (<0.40), fair (0.40 to 0.59), good (0.60 to 0.74), and excellent (0.75 to 1.00) agreement, according to Cicchetti (1994). To determine differences between operators (i.e., interoperator reproducibility) and duplicate scans (i.e., intraoperator repeatability) for the proximal, middle, and distal aspects of the teat, respectively, we fitted 3 general linear mixed models with the “nlme” package (Pinheiro et al., 2016). To account for dependence of images among different operators, we included image as a random effect and modeled the covariance of repeated measurements (i.e., duplicate scans) with the first-order autoregressive covariance structure. Operator and duplicate scans were forced into the models as fixed effects. Least squares means (LSM) and 95% CI were calculated with the “lsmeans” package (Lenth, 2016). We declared significance at *P* < 0.05.

We obtained 80 thermographic images (20 cows × 2 operators × 2 images) that resulted in a total of 480 measurements obtained from the 3 different aspects of the left and right hind teats. The overall mean ± SD (range) teat skin temperatures were proximal aspect, 33.2 ± 1.9°C (28.4–36.1°C); middle aspect, 32.4 ± 2.5°C (26.2–35.6°C); and distal aspect, 30.9 ± 3.4°C (21.6–35.1°C). Results for assessment of interoperator reproducibility and intraoperator repeatability are shown in Table 1. Pearson correlation coefficients indicated a very strong correlation between operators (r ≥0.95) and between duplicate scans within operators (r ≥0.94) for measurements at all 3 aspects. Similarly, ICC and CCC values revealed excellent interoperator reproducibility and intraoperator repeatability. Our data suggest that infrared thermography facilitates precise measurements of skin temperature of cows' hind teats. Values of r, ICC, and CCC obtained in this study were higher than those reported in previous studies evaluating the precision of ultrasound-based measurements of teat canal dimensions (Wieland et al., 2018) and measurements of teat blood perfusion with power Doppler ultrasonography (Wieland et al., 2019). The variability in measurements of teat skin temperature using infrared thermography may be attributable to (1) biological variation, (2) differences between thermographic images, and (3) discrepancies between measurements using the adjunct software program. Maltz et al. (2000) reported that teat muscle contractions that can occur after tactile stimulation decrease the arterial blood flow of the teat and influence its dimensions. The noncontact nature of thermographic imaging likely diminished the frequency of teat muscle contractions, minimizing the biological variation of teat skin temperature that could have occurred at a larger scale with sensors that require physical contact with the teat. Second, we attempted to reduce variability between images by using a standardized scanning technique, including constant imaging modes and distance between the camera and the udder. Further, both operators were trained on all procedures before the study, which aided in timely acquisition of high-quality images and likely reduced variability between images. Last, we believe that image analysis by a single operator following a standard operating procedure, as well as using the average temperature as opposed to single measurements, reduced measurement error during the image analyses.

**Table 1:** Pearson correlation coefficients (r), intraclass correlation coefficients (ICC), and concordance correlation coefficients (CCC) for assessment of interoperator reproducibility and intraoperator repeatability, as well as least squares means (LSM) and 95% CI for each operator and duplicate thermograms^1^

Item	Interoperator reproducibility			Intraoperator repeatability		
	Proximal	Middle	Distal	Proximal	Middle	Distal
r (95% CI)^2^	0.95 (0.91—0.97)	0.99 (0.97—0.99)	0.99 (0.97—0.99)	0.94 (0.91—0.96)	0.99 (0.98—0.99)	0.99 (0.98—0.99)
ICC (95% CI)^3^	0.95 (0.91—0.97)	0.98 (0.97—0.99)	0.98 (0.97—0.99)	0.94 (0.92—0.96)	0.98 (0.98—0.99)	0.99 (0.98—0.99)
CCC (95% CI)^3^	0.95 (0.91—0.97)	0.98 (0.96—0.99)	0.98 (0.97—0.99)	0.94 (0.90—0.96)	0.99 (0.98—0.99)	0.99 (0.98—0.99)
ANOVA^4^						
OP1	33.2 (32.6—33.8)	32.4 (31.5—33.2)	30.9 (29.8—32.0)			
OP2	33.2 (32.6—33.8)	32.4 (31.6—33.3)	31.0 (29.9—32.0)			
*P*-value	0.95	0.14	0.20			
DUP1				33.3 (32.7—33.9)	32.5 (31.7—33.3)	31.0 (29.9—32.1)
DUP2				33.2 (32.6—33.8)	32.3 (31.5—33.1)	30.8 (29.7—31.9)
*P*-value				0.20	0.0001	<0.0001

1 Results were derived from mixed-effects ANOVA for the average teat skin temperature (°C) measured at the proximal, middle, and distal aspects of the left and right hind teats in 20 dairy cows using a thermography camera.

2 Classification system for Pearson correlation coefficients according to Schober et al. (2018): negligible, 0.00 to 0.09; weak, 0.10 to 0.39; moderate, 0.40 to 0.69; strong, 0.70 to 0.89; and very strong, 0.90 to 1.00.

3 Classification system for ICC and CCC according to Cicchetti (1994): poor, <0.40; fair, 0.40 to 0.59; good, 0.60 to 0.74; excellent, 0.75 to 1.00.

4 Mixed-effects ANOVA including the fixed effects of operator (OP1, operator 1; OP2, operator 2) and duplicate measurements (DUP1, first thermogram; DUP2, second thermogram). LSM (95% CI) and *P*-value for the fixed effect of operator and duplicate measurement, respectively, are shown.

The general linear mixed models revealed that the numerical differences in average teat skin temperature at the proximal, middle, and distal aspects of the teat between operators were likely due to chance (*P* ≥ 0.14). No meaningful differences were detected for duplicate measurements of the average teat skin temperature at the proximal aspect of the teat (*P* = 0.20). Conversely, we observed differences between duplicate scans for measurements at the middle and distal aspects (*P* ≤ 0.0001). However, although these differences were statistically significant, we should consider their magnitude rather than the *P*-value alone, which is affected by the number of observations in the study. The clinical significance of these differences is the subject of future investigation. Our ultimate goal is to assess, via teat skin temperature measurements, alterations in the teats' circulatory system that occur because of mechanical forces during machine milking. The ability to detect an effect of interest depends on its size relative to the variance of the residual error (Lin, 1989). The data obtained in this study will help in estimation of sample size calculations for future studies. We conclude that precise measurements of skin temperature of cows' hind teats can be obtained with infrared thermography. Thermography can be applied in the future to assess the effects of different milking machine settings (e.g., vacuum, pulsation, automatic cluster remover, and milking liner) and different premilking stimulation strategies on teat blood circulation and associated teat defense mechanisms. These studies may contribute to further understanding of the interrelationships between the milking machine, the milking personnel, and the cow to help optimize the milk harvesting process.
